# Effect of vitamin D deficiency on clinical pregnancy outcomes in women with polycystic ovary syndrome undergoing *in vitro* fertilization

**DOI:** 10.3389/fendo.2025.1710004

**Published:** 2025-11-24

**Authors:** Lan Yu, Bin Hu, Jing Kong, Xiaohang Xu, Cuilian Zhang

**Affiliations:** 1 Reproductive Medicine Center, Henan Provincial People’s Hospital, Zhengzhou, China; 2 Reproductive Medicine Center, People’s Hospital of Zhengzhou University, Zhengzhou, China; 3 Department of Obstetrics and Gynecology, The Central Hospital of Wuhan, Tongji Medical College, Huazhong University of Science and Technology, Wuhan, China

**Keywords:** vitamin D, PCOS (polycystic ovary syndrome), infertility, IVF (*in vitro* fertilization), fresh embryo transfer (ET)

## Abstract

**Purpose:**

This study aimed to investigate the effect of serum vitamin D levels on *in vitro* fertilization and embryo transfer (IVF-ET) in patients with polycystic ovary syndrome (PCOS).

**Methods:**

This retrospective cohort study included patients with PCOS who underwent IVF therapy and received fresh embryo transfer for the first time. The enrolled cohort was divided into two groups based on serum 25-hydroxyvitamin D (25(OH)D) levels: Vitamin D deficiency (25(OH)D < 20 ng/mL) and vitamin D replete-insufficiency (25(OH)D ≥ 20 ng/mL). The primary outcome was clinical pregnancy. The secondary outcomes were the number of oocytes retrieved, MII oocytes, fertilized embryos, available embryos, high-quality embryos, blastocysts formed, and live birth rates.

**Results:**

This study included 613 patients who underwent their first IVF-ET cycle. Clinical pregnancy rates were significantly lower in patients with PCOS who had vitamin D deficiency than in those who had vitamin D replete-insufficiency [58.3% (211/367) versus 67.1% (163/246); *P* = 0.029]. Logistic regression, adjusted for endometrial thickness, progesterone, and vitamin D levels, demonstrated that serum vitamin D ≥ 20 ng/mL was independently associated with higher clinical pregnancy rates than the vitamin D deficiency group (odds ratio 1.48; 95% confidence interval, 1.02–2.32; *P* = 0.032). However, vitamin D deficiency did not significantly affect live birth rates (*P* = 0.57). We found no significant differences in the number of oocytes, MII oocytes, fertilized embryos, and the percentage of top-quality embryos between the two groups.

**Conclusion:**

This study suggests that vitamin D deficiency leads to lower clinical pregnancy rates in patients with PCOS undergoing IVF-ET. Furthermore, the serum vitamin D level is independently associated with clinical pregnancy rates in patients with PCOS undergoing IVF-ET.

## Introduction

Polycystic ovary syndrome (PCOS), with a prevalence of 5%–20% in women of reproductive age, is a common reproductive endocrine disorder and a leading cause of female infertility (1, 2). PCOS commonly presents with a combination of oligo-anovulation, hyperandrogenism, and polycystic ovarian morphology. A subset of affected individuals also exhibits concomitant metabolic disturbances, including insulin resistance, dyslipidemia, and broader metabolic dysfunction. Anovulation is a major cause of infertility in women with PCOS. Even when ovulatory cycles occur, these patients are at an elevated risk of infertility and miscarriage, potentially due to compromised oocyte quality and/or endometrial receptivity (3).

The precursor of vitamin D is synthesized in the skin upon exposure to sunlight. It is then sequentially hydroxylated in the liver and kidneys to form its active form, calcitriol (1,25-dihydroxyvitamin D), which mediates its genomic effects by binding to the vitamin D receptor (VDR). Gene for VDR is located on chromosome 12. Active VDR can regulate the target gene expression in many cell types, including those of the immune system. VDRs are present in calcium-regulating tissues and throughout the reproductive system, including the ovary, endometrium, placenta, and decidua in females as well as in the testes of males (4–6). Evidence indicates that vitamin D regulates key reproductive processes, including decidualization, implantation, and human chorionic gonadotropin (hCG) secretion (7). VDR expression in reproductive organs suggests a possible regulatory role for vitamin D in female reproductive physiology. Animal studies have demonstrated that compromised VDR expression correlates with impaired folliculogenesis, uterine hypoplasia, and reproductive impairments, including infertility and pregnancy complications (8–10). Moreover, clinical studies indicated that low vitamin D level is correlated with significantly higher risks of gestational diabetes, preeclampsia, recurrent pregnancy loss, and delivering small-for-gestational-age infants (11, 12).

Vitamin D deficiency is significantly more prevalent in patients with PCOS than in healthy controls and has been correlated with insulin resistance (IR), obesity, and metabolic syndrome (13, 14). Women with PCOS and vitamin D deficiency have lower ovulation and higher pregnancy loss (15, 16). Vitamin D supplementation improves reproductive outcomes, including the menstrual cycle, follicular development, and pregnancy rates in this population (14). Supporting evidence has also indicated that vitamin D levels affect IVF outcomes in patients with PCOS (17). Conversely, other studies have demonstrated no statistically significant correlation between pregnancy rates and serum or follicular 25(OH)D levels in women who underwent IVF (18).

However, emerging evidence has suggested a potential link between vitamin D status and reproductive outcomes (11, 19–22). Vitamin D deficiency is implicated in PCOS pathogenesis, though its causal role remains unclear (23). While it may increase live birth rates in PCOS patients (1, 24) and improve IVF outcomes in those with insulin resistance (15, 25), it is unknown if this is due to vitamin D’s metabolic improvements or direct effects on reproductive system. Conversely, high-dose supplementation of vitamin D may impair follicular development (14). The heterogeneity of PCOS itself, coupled with variations in clinical interventions and geographic differences, may account for the divergent conclusions reported across studies. The objective of this study was to investigate whether vitamin D levels impact pregnancy outcomes in infertile PCOS patients undergoing IVF.

## Materials and methods

### Study cohort

This retrospective cohort study included infertile women with PCOS. The patients underwent their first IVF cycle at the Henan Provincial People’s Hospital between June 2018 and December 2022. This study was included among 2,281 with PCOS who underwent their first IVF cycle at our center. A total of 613 patients were included in the final analysis. The inclusion criteria were as follows: (1) Patients diagnosed with PCOS following the 2023 updated Rotterdam diagnostic criteria, and (2) undergoing IVF stimulation and receiving fresh embryo transfer for the first time. The exclusion criteria were as follows: (1) Congenital or acquired uterine anomalies, (2) untreated hydrosalpinx, and (3) progesterone > 2.0 ng/mL on hCG trigger day. From the patients identified in the hospital database and screened for eligibility, 613 patients were included in the study. The study protocol was approved by the Reproductive Ethics Committee of Henan Provincial People’s Hospital (SYSZLL-2019110401).

### Patient data collection

In this study, the following patient information was collected: age, duration of infertility, type of infertility, body mass index (BMI), fasting insulin (FINS), fasting glucose (FGP), and homeostasis model assessment of insulin resistance (HOMA-IR), was collected. Baseline data, including measurements of basic follicle-stimulating hormone (FSH), luteinizing hormone (LH), estradiol (E2), progesterone (P), prolactin (PRL), testosterone (T), and anti-Müllerian hormone (AMH) levels, antral follicle count (AFC), and basal endometrial thickness, were collected. Ovarian stimulation characteristics and embryological outcomes were collected, including the controlled ovarian stimulation regimen, gonadotropin (Gn) dosage, number of retrieved oocytes, MII oocytes, available embryos, top-quality embryos, fertilization embryos. Progesterone levels and endometrial thickness were assessed on both the hCG trigger and embryo transfer days.

According to the clinically accepted ranges for vitamin D deficiency (26, 27) (< 20 ng/mL), participants who completed the study were divided into the following two groups: (1) vitamin D deficiency (25(OH)D < 20 ng/mL) and (2) vitamin D replete-insufficiency group (25(OH)D ≥ 20 ng/mL).

Each embryo was graded based on developmental speed, degree of fragmentation, and the evenness of the cleavage sphere. Embryos with 7–9 blastomeres, uniform cytoplasm, regular morphology, and < 10% fragmentation were considered high-quality embryos.

The primary outcome was the CPR, defined as the presence of an intrauterine sac with a gestational sac and fetal pole, and cardiac activity, at 28 and 35 days after embryo transfer. The secondary outcomes were the number of oocytes retrieved, MII oocytes, fertilized embryos, available embryos, high-quality embryos, blastocyst formation, and live birth rate. Serum vitamin D levels were measured before the patients entered IVF cycles using electrochemiluminescence. All patients underwent IVF-ET cycles using standardized regimens for pituitary downregulation and controlled ovarian hyperstimulation. The initial Gn dose (Gonal-f; EMD Serono, Geneva, Switzerland; 100–300 IU/day) was determined based on age, baseline FSH levels, and AFC, and subsequently adjusted according to ovarian response. Ovarian monitoring involved serial transvaginal ultrasonography and serum measurements of FSH, LH, E2, and P. When two to three follicles reached or exceeded 17/18 mm, hCG was administered. The hCG dose ranged from 4,000 to 10,000 IU based on the clinician’s concerns regarding ovarian hyperstimulation syndrome (OHSS). Transvaginal ultrasound-guided oocyte retrieval was performed 36–38 h following hCG injection. Fertilization was achieved via conventional insemination or intracytoplasmic sperm injection (ICSI). Fresh embryos were transferred under ultrasound guidance on day 3 or 5 after oocyte retrieval, if the endometrial thickness was ≥ 8 mm and the endometrium had homogeneous echogenicity. The number of embryos transferred during each procedure was determined based on the developmental stage of the embryos and the total number of viable embryos available for transfer. In fresh embryo transfer cycles, luteal phase support was initiated on the day of oocyte retrieval, consisting of oral dydrogesterone tablets twice a day (Duphaston, 10 mg per tablet, produced by Solvay Pharmaceuticals, Netherlands) in combination with 8% progesterone vaginal sustained-release gel once daily at a dose of 90 mg (Crinone, 90 mg per tube, manufactured by Merck Serono). Serum β-hCG levels > 50 IU/L were diagnosed as biochemical pregnancy 14 days after embryo transfer. Ultrasound examinations were performed 28 and 35 days after embryo transfer to define clinical pregnancy and rule out ectopic pregnancy.

### Data analysis

Statistical analysis was performed using the Statistical Package for the Social Sciences (SPSS) software (version 20.0; IBM Corp., Armonk, NY, USA). Continuous variables are presented as means, and categorical variables are presented as frequencies. The independent-samples T test and Mann–Whitney U test were used to assess normal and skewed distributional variables, respectively. Univariate and multivariate logistic regression analyses were performed to explore the effects of vitamin D on CPRs. Statistical significance was defined as *P* < 0.05.

## Results

### Patients’ baseline and ovarian stimulation characteristics

**Figure 1:**
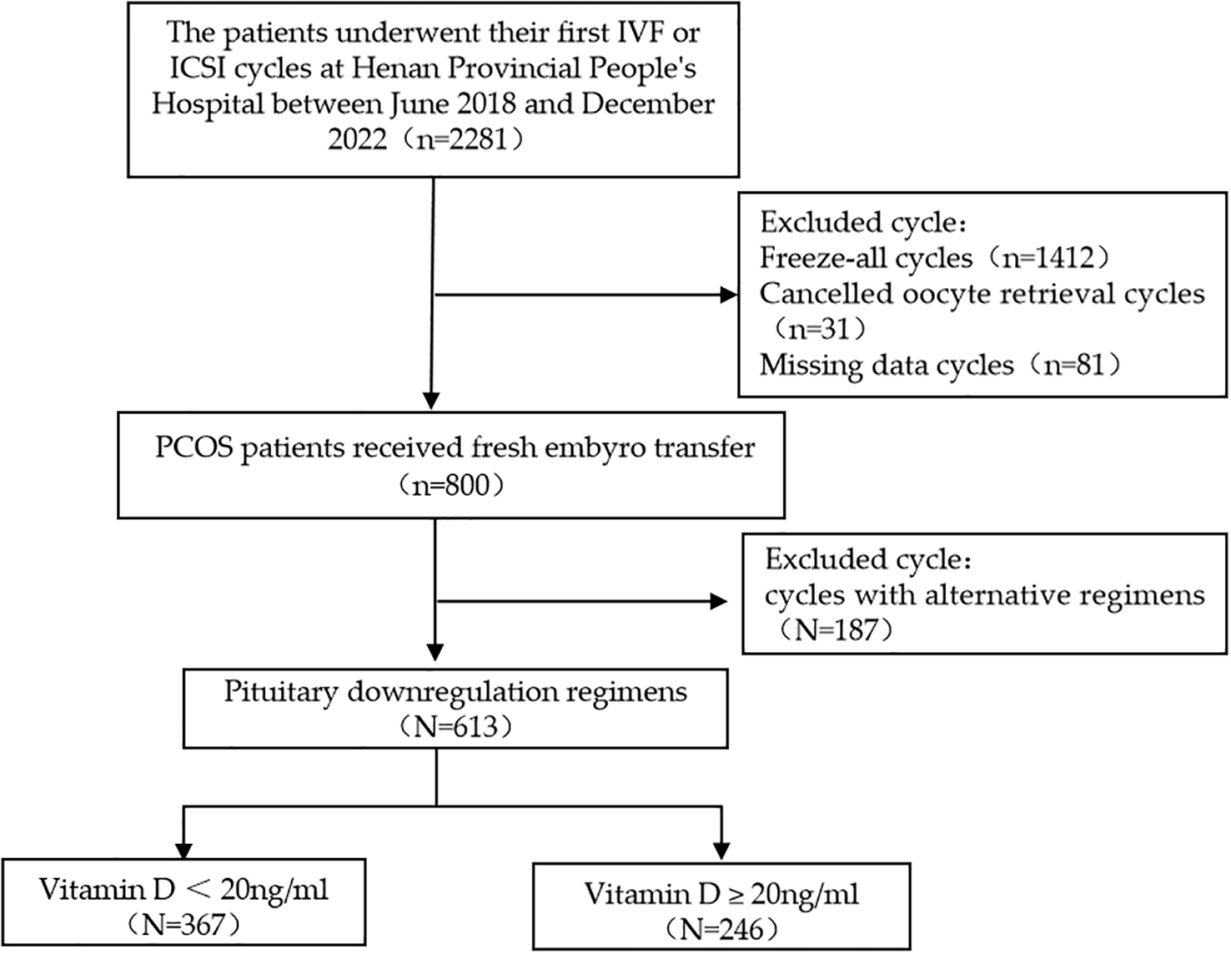
Flow diagram depicting the participant screening and selection process.

According to the inclusion and exclusion criteria, 613 patients were enrolled in this study (**Figure 1**). The baseline characteristics of the study population are presented in **Table 1**. Serum 25(OH)D levels were < 20 ng/mL in 367 women (59.9%) and ≥ 20 ng/mL in 246 women (40.1%). Baseline clinical characteristics, including age, BMI, duration of infertility, AMH, type of infertility, FGP, FINS, and HOMA-IR, were compared between women with vitamin D deficiency and those with 25(OH)D levels ≥ 20 ng/mL (**Table 1**). We found no statistically significant differences in Gn dose, duration of Gn treatment, and endometrial thickness on the day of hCG trigger between the two groups.

**Table 1:** Baseline characteristics of the study cohort.

Item	Vitamin D < 20ng/mL	Vitamin D ≥ 20ng/mL	P-value
	(n=367)(59.9%)	(n=246)(40.1%)	
Age, mean (SD)	29.7 ± 4.1	29.5 ± 3.7	0.60
BMI(kg/m2)	26.1 ± 4.1	25.8 ± 3.7	0.41
Years of infertility (year)	3.91 ± 2.74	4.08 ± 2.64	0.43
Type of infertility, n (%)			
Primary infertility	225/367(61.3%)	127/246(51.6%)	0.017
Secondary infertility	142/367(38.7%)	119/246(48.4%)	
Fertilization type			0.37
conventional insemination	314/367(85.6%)	221/246(89.8%)	
ICSI	53/367(14.4%)	25/246(10.2%)	
Vitamin D (ng/ml) (SD)	14.42 ± 3.20	23.46 ± 4.50	0.000
AMH(ng/L)	6.9 ± 3.9	7.3 ± 4.1	0.24
Basal FSH(IU/L)	5.96 ± 1.47	6.03 ± 1.35	0.57
Basal LH(IU/L)	8.32 ± 4.85	8.83 ± 5.06	0.21
Basal PRL(ng/mL)	14.67 ± 7.57	14.32 ± 6.41	0.59
Basal E2(pg/mL)	37.99 ± 17.04	42.42 ± 17.19	0.002
Basal T(ng/mL)	0.4 ± 0.21	0.42 ± 0.2	0.35
Basal P(ng/mL)	0.25 ± 0.2	0.24 ± 0.18	0.79
Fasting glucose (mg/dL)	4.97 ± 0.88	4.94 ± 0.82	0.59
Fasting insulin (mIU/mL)	18 ± 11.51	16.93 ± 9.27	0.23
HOMA-IR	4.06 ± 2.99	3.86 ± 2.65	0.42
AFC	22.62 ± 3.59	22.67 ± 4.08	0.89

BMI, body mass index; FSH, follicle stimulating hormone; LH, luteinizing hormone; E2, estradiol; AFC, antral follicle count; AMH, anti-Müllerian hormone; P, progesterone; ICSI, intracytoplasmic sperm injection;P ≤ 0.05 was considered statistically significant.

### Embryological and clinical outcomes

We observed no significant differences between vitamin D deficiency (25(OH)D < 20 ng/mL) and vitamin D replete-insufficiency (serum 25(OH)D ≥ 20 ng/mL) groups regarding the number of oocytes, MII oocytes, fertilized embryos, available embryos, number of high-quality embryos, and blastocyst formation (**Table 2**).

**Table 2:** Ovarian stimulation characteristics and embryological outcomes.

Item	Vitamin D < 20ng/mL	Vitamin D ≥ 20ng/mL	P
	(n=367) (59.9%)	(n=246)(61.1%)	
Gn dose(IU)	2565.48 ± 1345.59	2465 ± 1136.33	0.34
E2 on the day of hCG(pg/mL)	1776.43 ± 912.30	1750.57 ± 774.25	0.73
Endometrial thickness on the day of hCG(mm)	10.74 ± 2.23	10.87 ± 2.49	0.52
Number of oocytes retrieved, mean (SD)	10.18 ± 4.27	10.42 ± 4.22	0.48
Number of MII oocytes, mean (SD)	8.7 ± 3.93	8.9 ± 3.84	0.48
Number of fertilization embryos (SD)	6.07 ± 3.22	6.22 ± 3.07	0.54
Number available embryos (SD)	4.97 ± 2.90	5.16 ± 2.83	0.42
Number of high-quality embryos D3 (SD)	2.71 ± 2.33	2.72 ± 2.12	0.92
Number of blastocyst formation	2.96 ± 3.17	2.72 ± 2.68	0.31

Gn, gonadotropin; HCG, human chorionic gonadotropin; P ≤ 0.05 was considered significant.

### Pregnancy outcomes

CPRs were significantly lower in patients with PCOS who had vitamin D deficiency than in those who had vitamin D replete-insufficiency [58.3% (211/367) versus 67.1% (163/246); *P* = 0.029; **Table 3**]. However, vitamin D deficiency did not significantly affect live birth rates (*P* = 0.58). The incidence of ectopic pregnancy did not differ between the two groups (*P* = 0.88). Additionally, there were no significant differences in the number or type of embryo transfers between the two groups. Logistic regression analysis was performed, incorporating serum vitamin D level, endometrial thickness on the day of embryo transfer, and progesterone level on the day of hCG administration. After adjusting for these covariates, a serum 25(OH)D level ≥ 20 ng/mL remained independently associated with increased CPR (**Table 4**). Specifically, the odds of achieving clinical pregnancy were 1.48 times higher in the vitamin D replete-insufficiency group than in the deficiency group (odds ratio [OR], 1.48; 95% confidence interval [CI], 1.02–2.32; *P* = 0.032).

**Table 3:** Clinical outcomes.

Item	Vitamin D < 20ng/ml	Vitamin D ≥ 20ng/ml	P-value
	(n=367) (59.9%)	(n=246)(40.1%)	
Number of transferred embryos	1.38 ± 0.486	1.41 ± 0.414	0.37
Endometrial thickness on the transfer day (mm)	10.87 ± 2.51	10.89 ± 2.57	0.91
Type of transferred embryos			
D3 cleavage-stage embryo	281 (76.6%)	175 (71.1%)	0.13
D5 blastocyst	86 (23.4%)	71 (28.9%)	
Clinical pregnancy, n (%)	211(367) 58.3%	163(246) 67.1%	0.029
Ectopic pregnancy rate (%)	5 (367) 1.4%	3 (243) 1.2%	0.88
Live birth, n (%)	184(367) 50.1%	129(246) 51.1%	0.58

**Table 4:** Logistic regression analysis.

Item	Unadjusted			Adjusted		
	OR	95%CI	P	OR	95%CI	P
Vitamin D < 20ng/ml				Ref		
Vitamin D ≥ 20ng/ml	1.50	1.05∼2.15	0.025	1.48	1.03∼2.12	0.032
P on the day of hCG(ng/mL)	2.11	1.04∼4.26	0.037	2.12	1.04∼4.32	0.039
Endometrial thickness on the transfer day (mm)	1.08	1.01∼1.16	0.031	1.09	1.01∼1.16	0.023

Furthermore, logistic regression analysis revealed that endometrial thickness on the day of transfer and progesterone levels on the day of hCG administration were independently associated with CPRs (**Table 4**).

## Discussion

In this cohort of women with PCOS who underwent IVF-ET treatment, vitamin D deficiency (serum 25(OH)D level < 20 ng/mL) was observed in 59.9% of the participants. This analysis revealed significantly reduced pregnancy rates among women with vitamin D deficiency compared to those with replete-insufficiency. Vitamin D levels did not affect IVF laboratory characteristics.

Vitamin D deficiency is a globally recognized condition globally. Research has indicated a correlation between vitamin D deficiency and an elevated risk of hypertension, infectious diseases, autoimmune diseases, and reproductive system disorders, including preeclampsia, miscarriage, and infertility (28). Halloran et al. reported that lower vitamin D levels impaired the fertility rate and affected neonatal growth in rats (8). Studies have revealed that vitamin D deficiency is more prevalent in PCOS, and lower vitamin D levels are a risk factor for PCOS development (10, 29–31). The association between vitamin D levels and IVF-ET outcomes, particularly CPRs, has been examined in several studies, with inconsistent evidence (10, 21, 32). Despite the ongoing controversy regarding the impact of vitamin D on assisted reproductive technology outcomes, this study investigated its role in infertile patients with PCOS by comparing fresh-cycle pregnancy rates across varying vitamin D levels.

Compromised vitamin D levels in PCOS have been associated with elevated AMH levels and metabolic abnormalities, such as elevated HOMA-IR, T level, and obesity (5, 33, 34). Additionally, vitamin D supplementation has been demonstrated to reduce AMH levels, thereby potentially enhancing folliculogenesis in PCOS (3, 33, 35). In this study, we found no association between vitamin D level and AMH, HOMA-IR, or BMI. Consistent with these research outcomes, a large retrospective investigation has indicated no correlation between AMH levels and vitamin D levels in patients with PCOS and the control group (23). Similarly, low serum vitamin D concentrations may be a consequence rather than the cause of obesity. It has been suggested that low levels of vitamin D and genes associated with reduced vitamin D concentrations exert a relatively minor influence on obesity (36). Vitamin D supplementation in women with PCOS could decrease the fasting glucose, HOMA-IR, and T level (37, 38). In this study, the two groups did not exhibit significant differences in HOMA-IR and T level. These findings are consistent with those of Bostanci et al. and Arslan et al. (23, 39). These non-significant differences may be attributed to the low vitamin D levels in both groups. Further studies are needed to determine whether additional vitamin D supplementation affects these indicators.

Studies have indicated that women with vitamin D deficiency and PCOS exhibit reduced ovulation rates during ovarian stimulation cycles (17, 19). This effect can be reversed with supplementation and potentially mediated by inducing vitamin D upregulation of the soluble receptor for advanced glycation end products, which binds circulating AGEs and inhibits their inflammatory deleterious effects in PCOS (13, 15, 40–42). Adequate vitamin D status is also associated with improved fertilization rates during IVF in women with PCOS (17). To date, the direct effect of vitamin D on the quality of human oocytes and embryos has not been investigated. In this study, the two groups shared a similar number of mature oocytes, 2PN embryos, available cleavage embryos, and blastocysts. These data indicate that vitamin D has no effect of vitamin D on egg maturation, fertilization rate, and embryo development. In slim PCOS patients, 25(OH)D_3_ correlated with embryo fertilization rates. However, 25(OH)D_2_ was associated with improved cleavage rate, top-quality day-3 embryos, and blastocyst formation in both PCOS and non-PCOS groups (17). This result suggests that the biological effects of different metabolites of vitamin D may vary across various systems, and their differential effects in the ovary require further clarification (17). The vitamin D levels detected in our study contained both 25(OH)D_3_ and 25(OH)D_2_. The varying conclusions across studies can be attributed to the specific vitamin D metabolite examined.

This study found no significant difference between vitamin D deficiency (< 20 ng/mL) and replete-insufficiency (≥ 20 ng/mL) groups in the number of embryos transferred or the type of embryos transferred. Likewise, endometrial thickness on the day of embryo transfer and progesterone levels on the day of hCG trigger were comparable between the two groups. However, CPR was significantly higher in the vitamin D replete-insufficiency group, suggesting that despite similar embryo quality and endometrial thickness, these patients may exhibit more favorable endometrial receptivity for embryo implantation. This aligns with studies that have reported a similar association between high vitamin D levels and improved reproductive outcomes. Sufficient serum levels of vitamin D (25(OH)D ≥ 30 ng/mL or 20 ng/mL) have been associated with higher CPRs in recipients of oocyte donation than in those with lower vitamin D levels (25(OH)D < 30 ng/mL or 20 ng/mL) (13, 43). Some studies have suggested that vitamin D may influence IVF outcomes by critically regulating endometrial homeobox 10 (*HOXA10*) expression (44). The *HOXA10* gene is a key regulator of endometrial decidualization and immunomodulation essential for embryo implantation (45). A study reported that endometrial HOXA10 mRNA expression was significantly higher in women with PCOS who had sufficient serum vitamin D levels than in those with low serum vitamin D levels (44, 45). Besides its effect on HOXA10, vitamin D also plays an immunomodulatory role. In *in vitro* trophoblast experiments, vitamin D deficiency decreased the expression of hCG and increased the expression of tumor necrosis factor-alpha, interleukin-6, and interferon-gamma, thereby increasing the T-helper/T-cytotoxic cell ratio (46). Vitamin D deficiency is also associated with an increased prevalence of autoantibodies, including antiphospholipid antibodies, anti-thyroperoxidase antibodies, antibodies to nuclear antigens, and anti-ssDNA antibodies (47). Therefore, the higher pregnancy rate with vitamin D levels > 20 ng/mL may be partly due to the effect of vitamin D on endometrial receptivity.

In this study, we did not observe significant differences between the two groups in terms of basic metabolic parameters, such as BMI or fasting insulin levels, nor in embryonic data. Given the disparity in CPR between the two groups, we speculate that the underlying cause may be endometrial receptivity. The effect of vitamin D on endometrial receptivity can be determined only through an extensive combination of basic and clinical studies.

This study has several limitations. Maternal serum vitamin D levels were not monitored longitudinally until delivery, and potential vitamin D supplementation during this period was not assessed. Consequently, we did not draw definitive conclusions regarding the live birth rates. As a result, the findings regarding live birth rates should be interpreted cautiously. As this was a single-center retrospective analysis we cannot rule out the possibility of residual or unmeasured confounding factors, such as dietary habits, specific level of sun exposure, or using of supplements which could influence both vitamin D status and reproductive outcomes Moreover, studies have implied that vitamin D status is not a uniform condition, but is likely driven by distinct vitamin D metabolites, each exerting unique biological effects. This variability may explain the inconsistent findings and debates regarding the consequences of deficiency and the response to supplementation. Vitamin D measurement methods vary across studies, and vitamin D-binding protein polymorphisms have not been considered. Therefore, the relationship between vitamin D levels and pregnancy rate should be interpreted more carefully. These data indicate that vitamin D deficiency may not be a uniform condition, potentially influenced by various vitamin D metabolites and their resultant effects. This variability could explain the heterogeneity and ongoing debate regarding the impact of vitamin D deficiency and the efficacy of supplementation.

## Data Availability

The original contributions presented in the study are included in the article/supplementary material. Further inquiries can be directed to the corresponding author.

## References

[1] Pal L Zhang H Williams J Santoro NF Diamond MP Schlaff WD . Vitamin D status relates to reproductive outcome in women with polycystic ovary syndrome: secondary analysis of a multicenter randomized controlled trial. J Clin Endocrinol Metab. (2016) 101:3027–35. doi: 10.1210/jc.2015-4352, PMID: 27186859 PMC4971341

[2] Azziz R . Polycystic ovary syndrome. Obstet Gynecol. (2018) 132:321–36. doi: 10.1097/AOG.0000000000002698, PMID: 29995717

[3] Palomba S Daolio J La Sala GB . Oocyte competence in women with polycystic ovary syndrome. Trends Endocrinol Metab. (2017) 28:186–98. doi: 10.1016/j.tem.2016.11.008, PMID: 27988256

[4] Muscogiuri G Altieri B De Angelis C Palomba S Pivonello R Colao A . Shedding new light on female fertility: The role of vitamin D. Rev Endocr Metab Disord. (2017) 18:273–83. doi: 10.1007/s11154-017-9407-2, PMID: 28102491

[5] Tian M Zeng S Cai S Reichetzeder C Zhang X Yin C . 25(OH)VitD and human endocrine and functional fertility parameters in women undergoing IVF/ICSI. Front Endocrinol. (2022) 13:986848. doi: 10.3389/fendo.2022.986848, PMID: 36105399 PMC9464865

[6] Harkness LS Bonny AE . Calcium and vitamin D status in the adolescent: key roles for bone, body weight, glucose tolerance, and estrogen biosynthesis. J Pediatr Adolesc Gynecol. (2005) 18:305–11. doi: 10.1016/j.jpag.2005.06.002, PMID: 16202933

[7] Knabl J Vattai A Ye Y Jueckstock J Hutter S Kainer F . Role of placental VDR expression and function in common late pregnancy disorders. Int J Mol Sci. (2017) 18:2340. doi: 10.3390/ijms18112340, PMID: 29113124 PMC5713309

[8] Halloran BP Deluca HF . Effect of vitamin D deficiency on fertility and reproductive capacity in the female rat. J Nutr. (1980) 110:1573–80. doi: 10.1093/jn/110.8.1573, PMID: 7400847

[9] Kinuta K Tanaka H Moriwake T Aya K Kato S Seino Y . Vitamin D is an important factor in estrogen biosynthesis of both female and Male gonads*. Endocrinology. (2000) 141:1317–24. doi: 10.1210/endo.141.4.7403, PMID: 10746634

[10] Cozzolino M Busnelli A Pellegrini L Riviello E Vitagliano A . How vitamin D level influences *in vitro* fertilization outcomes: results of a systematic review and meta-analysis. Fertil Steril. (2020) 114:1014–25. doi: 10.1016/j.fertnstert.2020.05.040, PMID: 33012554

[11] Shahrokhi SZ Ghaffari F Kazerouni F . Role of vitamin D in female reproduction. Clin Chim Acta. (2016) 455:33–8. doi: 10.1016/j.cca.2015.12.040, PMID: 26747961

[12] Rostami M Tehrani FR Simbar M Bidhendi Yarandi R Minooee S Hollis BW . Effectiveness of prenatal vitamin D deficiency screening and treatment program: a stratified randomized field trial. J Clin Endocrinol Metab. (2018) 103:2936–48. doi: 10.1210/jc.2018-00109, PMID: 29788364

[13] Banker M Sorathiya D Shah S . Vitamin D deficiency does not influence reproductive outcomes of IVF-ICSI: a study of oocyte donors and recipients. J Hum Reprod Sci. (2017) 10:79. doi: 10.4103/jhrs.JHRS_117_16, PMID: 28904494 PMC5586094

[14] Menichini D Forte G Orrù B Gullo G Unfer V Facchinetti F . The role of vitamin D in metabolic and reproductive disturbances of polycystic ovary syndrome: a narrative mini-review. Int J Vitam Nutr Res. (2022) 92:126–33. doi: 10.1024/0300-9831/a000691, PMID: 33284035

[15] Zhao J Liu S Wang Y Wang P Qu D Liu M . Vitamin D improves *in-vitro* fertilization outcomes in infertile women with polycystic ovary syndrome and insulin resistance. Minerva Med. (2019) 110(2019):199–208. doi: 10.23736/S0026-4806.18.05946-3, PMID: 30612423

[16] Aghadavod E Mollaei H Nouri M Hamishehkar H . Evaluation of relationship between body mass index with vitamin D receptor gene expression and vitamin D levels of follicular fluid in overweight patients with polycystic ovary syndrome. Int J Fertil Steril. (2017) 11(2017):105–111. doi: 10.22074/ijfs.2017.4704, PMID: 28670428 PMC5347447

[17] Cunningham TK Allgar V Dargham SR Kilpatrick E Sathyapalan T Maguiness S . Association of vitamin D metabolites with embryo development and fertilization in women with and without PCOS undergoing subfertility treatment. Front Endocrinol. (2019) 10:13. doi: 10.3389/fendo.2019.00013, PMID: 30761082 PMC6361765

[18] Firouzabadi RD Rahmani E Rahsepar M Firouzabadi MM . Value of follicular fluid vitamin D in predicting the pregnancy rate in an IVF program. Arch Gynecol Obstet. (2014) 289:201–6. doi: 10.1007/s00404-013-2959-9, PMID: 23880888

[19] Hasan HA Barber TM Cheaib S Coussa A . Preconception vitamin D level and *In vitro* fertilization: pregnancy outcome. Endocr Pract. (2023) 29:235–9. doi: 10.1016/j.eprac.2023.01.005, PMID: 36642384

[20] Lv SS Wang JY Wang XQ Wang Y Xu Y . Serum vitamin D status and *in vitro* fertilization outcomes: a systematic review and meta-analysis. Arch Gynecol Obstet. (2016) 293:1339–45. doi: 10.1007/s00404-016-4058-1, PMID: 27022933

[21] Zhao J Huang X Xu B Yan Y Zhang Q Li Y . Whether vitamin D was associated with clinical outcome after IVF/ICSI: a systematic review and meta-analysis. Reprod Biol Endocrinol: RB&E. (2018) 16:13. doi: 10.1186/s12958-018-0324-3, PMID: 29426322 PMC5807754

[22] Polyzos NP Anckaert E Guzman L Schiettecatte J Van Landuyt L Camus M . Vitamin D deficiency and pregnancy rates in women undergoing single embryo, blastocyst stage, transfer (SET) for IVF/ICSI. Hum Reprod. (2014) 29:2032–40. doi: 10.1093/humrep/deu156, PMID: 24951484

[23] Arslan E Gorkem U Togrul C . Is there a relationship between vitamin D deficiency status and PCOS in infertile women? Geburtshilfe Frauenheilkd. (2019) 79:723–30. doi: 10.1055/a-0871-6831, PMID: 31303660 PMC6620183

[24] Butts SF Seifer DB Koelper N Senapati S Sammel MD Hoofnagle AN . Vitamin D deficiency is associated with poor ovarian stimulation outcome in PCOS but not unexplained infertility. J Clin Endocrinol Metab. (2019) 104:369–78. doi: 10.1210/jc.2018-00750, PMID: 30085176 PMC6300410

[25] Várbíró S Takács I Tűű L Nas K Sziva RE Hetthéssy JR . Effects of vitamin D on fertility, pregnancy and polycystic ovary syndrome—a review. Nutrients. (2022) 14:1649. doi: 10.3390/nu14081649, PMID: 35458211 PMC9029121

[26] Amrein K Scherkl M Hoffmann M Neuwersch-Sommeregger S Köstenberger M Tmava Berisha A . Vitamin D deficiency 2.0: an update on the current status worldwide. Eur J Clin Nutr. (2020) 74:1498–513. doi: 10.1038/s41430-020-0558-y, PMID: 31959942 PMC7091696

[27] Demay MB Pittas AG Bikle DD Diab DL Kiely ME Lazaretti-Castro M . Vitamin D for the prevention of disease: an endocrine society clinical practice guideline. J Clin Endocrinol Metab. (2024) 109:1907–47. doi: 10.1210/clinem/dgae290, PMID: 38828931

[28] Grant W Wimalawansa S Pludowski P Cheng R . Vitamin D: evidence-based health benefits and recommendations for population guidelines. Nutrients. (2025) 17:277. doi: 10.3390/nu17020277, PMID: 39861407 PMC11767646

[29] Bindayel IA . Low vitamin D level in saudi women with polycystic ovary syndrome. Front Nutr. (2021) 8:611351. doi: 10.3389/fnut.2021.611351, PMID: 33912581 PMC8072208

[30] Rudick B Ingles S Chung K Stanczyk F Paulson R Bendikson K . Characterizing the influence of vitamin D levels on IVF outcomes. Hum Reprod. (2012) 27:3321–7. doi: 10.1093/humrep/des280, PMID: 22914766

[31] Hu R Li L Liang L Qi Y Ma X Yang Y . 25(OH)D3 improves granulosa cell proliferation and IVF pregnancy outcomes in patients with endometriosis by increasing G2M+S phase cells. Reprod Biol Endocrinol. (2023) 21:115. doi: 10.1186/s12958-023-01165-8, PMID: 38053145 PMC10696887

[32] Chu J Gallos I Tobias A Tan B Eapen A Coomarasamy A . Vitamin D and assisted reproductive treatment outcome: a systematic review and meta-analysis. Hum Reprod (Oxf Engl). (2018) 33:65–80. doi: 10.1093/humrep/dex326, PMID: 29149263

[33] Dastorani M Aghadavod E Mirhosseini N Foroozanfard F Zadeh Modarres S Amiri Siavashani M . The effects of vitamin D supplementation on metabolic profiles and gene expression of insulin and lipid metabolism in infertile polycystic ovary syndrome candidates for *in vitro* fertilization. Reprod Biol Endocrinol. (2018) 16:94. doi: 10.1186/s12958-018-0413-3, PMID: 30286768 PMC6172745

[34] Ozkan S Jindal S Greenseid K Shu J Zeitlian G Hickmon C . Replete vitamin D stores predict reproductive success following *in vitro* fertilization. Fertil Steril. (2010) 94:1314–9. doi: 10.1016/j.fertnstert.2009.05.019, PMID: 19589516 PMC2888852

[35] Moridi I Chen A Tal O Tal R . The association between vitamin D and anti-müllerian hormone: a systematic review and meta-analysis. Nutrients. (2020) 12:1567. doi: 10.3390/nu12061567, PMID: 32481491 PMC7352921

[36] Vimaleswaran KS Berry DJ Lu C Tikkanen E Pilz S Hiraki LT . Causal relationship between obesity and vitamin D status: Bi-directional mendelian randomization analysis of multiple cohorts. PloS Med. (2013) 10:e1001383. doi: 10.1371/journal.pmed.1001383, PMID: 23393431 PMC3564800

[37] March WA Moore VM Willson KJ Phillips DIW Norman RJ Davies MJ . The prevalence of polycystic ovary syndrome in a community sample assessed under contrasting diagnostic criteria. Hum Reprod. (2010) 25:544–51. doi: 10.1093/humrep/dep399, PMID: 19910321

[38] Goodarzi MO Dumesic DA Chazenbalk G Azziz R . Polycystic ovary syndrome: etiology, pathogenesis and diagnosis. Nat Rev Endocrinol. (2011) 7:219–31. doi: 10.1038/nrendo.2010.217, PMID: 21263450

[39] Bostanci EI Ozler S Yilmaz NK Yesilyurt H . Serum 25-hydroxy vitamin D levels in turkish adolescent girls with polycystic ovary syndrome and the correlation with clinical/biochemical parameters. J Pediatr Adolesc Gynecol. (2018) 31:270–3. doi: 10.1016/j.jpag.2017.07.008, PMID: 28782659

[40] Garg D Grazi R Lambert-Messerlian GM Merhi Z . Correlation between follicular fluid levels of sRAGE and vitamin D in women with PCOS. J Assist Reprod Genet. (2017) 34:1507–13. doi: 10.1007/s10815-017-1011-6, PMID: 28825156 PMC5699991

[41] Li J Li M Li Y Zhao X Guan Y Zhang Y . Do serum vitamin D levels affect assisted reproductive outcomes and perinatal outcomes in young non-PCOS patients? A retrospective study. Arch Gynecol Obstet. (2024) 309:2099–106. doi: 10.1007/s00404-024-07410-8, PMID: 38429582

[42] Merhi Z . Advanced glycation end products and their relevance in female reproduction. Hum Reprod. (2014) 29:135–45. doi: 10.1093/humrep/det383, PMID: 24173721

[43] Rudick BJ Ingles SA Chung K Stanczyk FZ Paulson RJ Bendikson KA . Influence of vitamin D levels on *in vitro* fertilization outcomes in donor-recipient cycles. Fertil Steril. (2014) 101:447–52. doi: 10.1016/j.fertnstert.2013.10.008, PMID: 24210230

[44] Shilpasree A Kulkarni VB Shetty P Bargale A Goni M Oli A . Induction of endometrial HOXA 10 gene expression by vitamin D and its possible influence on reproductive outcome of PCOS patients undergoing ovulation induction procedure. Indian J Endocrinol Metab. (2022) 26:252–8. doi: 10.4103/ijem.ijem_90_22, PMID: 36248036 PMC9555374

[45] Ersahin SS Ersahin A . Serum 25-hydroxyvitamin D correlates with endometrial HOXA10 mRNA expression. Eur Rev Med Pharmacol Sci. (2022) 26:3483–6. doi: 10.26355/eurrev_202205_28842, PMID: 35647828

[46] Díaz L Noyola-Martínez N Barrera D Hernández G Avila E Halhali A . Calcitriol inhibits TNF-α-induced inflammatory cytokines in human trophoblasts. J Reprod Immunol. (2009) 81:17–24. doi: 10.1016/j.jri.2009.02.005, PMID: 19501915

[47] Ota K Dambaeva S Han A-R Beaman K Gilman-Sachs A Kwak-Kim J . Vitamin D deficiency may be a risk factor for recurrent pregnancy losses by increasing cellular immunity and autoimmunity. Hum Reprod. (2014) 29:208–19. doi: 10.1093/humrep/det424, PMID: 24277747

